# VEXAS: A review of current understandings and emerging treatment strategies

**DOI:** 10.3389/fimmu.2025.1644404

**Published:** 2025-07-28

**Authors:** Robert Holden, Yogeshraj Jeelall, Andrew McLean-Tooke, Kylan Pathmanathan, David Nolan

**Affiliations:** 1 Clinical Immunology, Royal Perth Hospital, Perth, WA, Australia; 2 Haematology, Royal Perth Hospital, Perth, WA, Australia; 3 Medical School, University of Western Australia, Perth, WA, Australia; 4 Clinical Immunology, Sir Charles Gairdner Hospital, Perth, WA, Australia; 5 Rheumatology, Sir Charles Gairdner Hospital, Perth, WA, Australia

**Keywords:** VEXAS syndrome, VEXAS, autoinflamatory diseases, myeloid cells, myelodyslastic syndromes

## Abstract

VEXAS (Vacuoles, E1 enzyme, X-linked, Autoinflammatory, Somatic) syndrome is a late-onset autoinflammatory disorder, typically affecting males, caused by somatic mutations in the X-linked gene UBA1 encoding the E1 ubiquitin-activating enzyme. These mutations result in defective ubiquitination and dysregulation of protein degradation, leading to Endoplasmic Reticulum stress and activation of innate immune pathways. This leads to significant inflammatory manifestations including fever, chondritis, neutrophilic dermatoses, and cytopenia’s and a range of inflammatory manifestations that define the clinical syndrome. Alongside these autoinflammatory manifestations, VEXAS exhibits features of clonal haematopoiesis, with clonal dominance of UBA1-mutant haematopoietic stem and progenitor cells with preferential myeloid differentiation and impaired generation of megakaryocytes, erythroid and lymphoid cells. The convergence of somatic mutation, inflammation, and bone marrow failure situates VEXAS at the interface of autoinflammation and hematologic neoplasia. Therapeutic approaches have focused on immunosuppression (e.g., corticosteroids, IL-6 inhibitors, JAK inhibitors), though these often yield only partial responses. Targeted therapies aimed at the mutant clone—including hypomethylating agents are under investigation. Allogeneic hematopoietic stem cell transplantation remains the only curative strategy. This review synthesises recent genetic, cellular, and clinical advances to consider VEXAS as an age-related proteosomopathy that unites clonal haematopoiesis with innate-immune dysregulation and provides appraisal of both established immunomodulators and emerging clone-directed therapies in addition to advocating harmonised response criteria, thereby offering a cohesive roadmap for future mechanistic studies and trial design in this rapidly evolving field.

## UBA1 in health and disease

**Figure 1:**
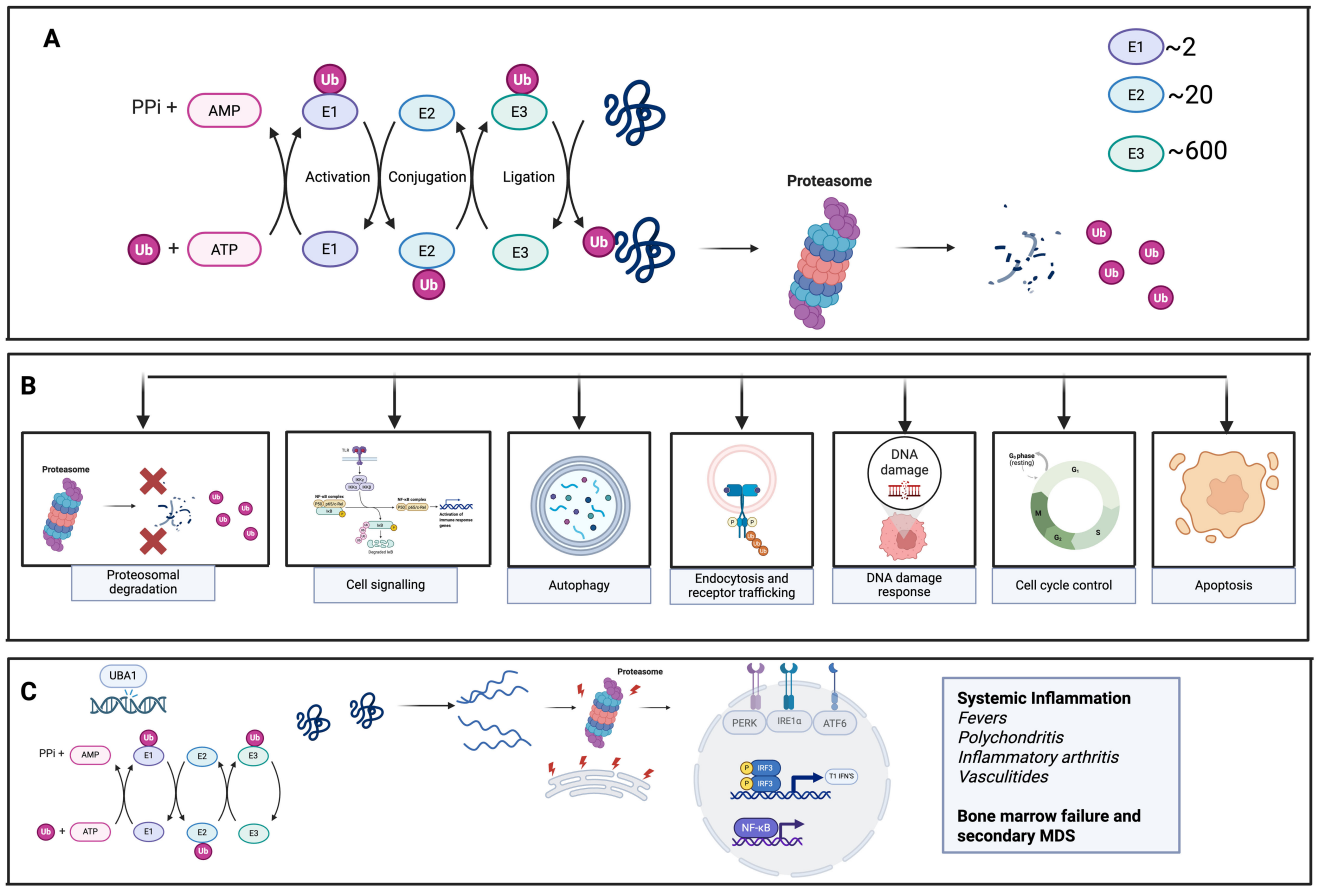
Ubiquitination in cellular processes and the results of pathogenic UBA1 mutation. **(A)** Demonstrates the normal process of Ubiquitination. Ubiquitin is attached to target proteins through a multi-step process involving three main enzymes: E1 (UBA1), E2, and E3 ligases. UBA1 activates ubiquitin, then transfers it to E2 enzymes, which, with the help of E3 ligases, facilitate the final transfer of ubiquitin to the substrate protein. The diversity of E3 ligases ensures specificity in this process. Once tagged with ubiquitin, proteins are recognized by proteasomes, which degrade them, playing a key role in regulating protein turnover and maintaining cellular homeostasis. **(B)** Normal functions of Ubiquitination in various cellular processes **(C)** UBA1 mutation results in reduction in UBA1 enzyme function, leading to failure of ubiquitination, activation of the unfolded protein response (UPR) due to endoplasmic reticulum (ER) stress and activation of proinflammatory cytokines and inflammation.

Ubiquitylation is a post-translational modification in which the small protein ubiquitin is covalently attached to target proteins. This process can tag proteins for degradation by the proteosome, alter their cellular location, affect their activity or influence their interactions with other proteins (1, 2). As a key regulatory mechanism, ubiquitylation plays essential roles in maintaining protein homeostasis, regulating the cell cycle, modulating immune responses and controlling various signalling pathways (3) (**Figures 1A, B**).

Ubiquitin is attached to its target proteins through a multi-step enzymatic process involving three main enzyme classes. (**Figure 1A**) The primary enzyme responsible for initiating this cascade- activating, conjugating and ligating ubiquitin to substrate proteins is the E1 ubiquitin-activation enzyme known as UBA1 (1). UBA1 first activates ubiquitin by binding it, along with ATP and Mg²^+^, at one of its adenylation domains, while its other domain helps stabilize the complex. The C-terminal glycine of ubiquitin is then adenylated, allowing UBA1’s active-site cysteine to form a thioester bond with ubiquitin. A second round of ATP- and Mg²^+^-dependent adenylation occurs, allowing a second ubiquitin to bind UBA1. This dual ubiquitin loading is thought to facilitate efficient transfer to downstream E2-conjugating enzymes by promoting a favourable conformational arrangement of the UBA1–ubiquitin complex (1, 3). The activated ubiquitin is then handed off to one of approximately 40 E2 enzymes via another thioester linkage. E3 ligases subsequently bring the E2–ubiquitin complex into proximity with the target protein, enabling ubiquitin transfer. Some E3 ligases, specifically those containing HECT domains, form a direct thioester intermediate with ubiquitin before transferring it to the substrate protein. The large diversity of E3 enzymes—numbering in the hundreds—provides specificity to the overall ubiquitination process (3).

This sequence of activation and transfer can repeat, resulting in polyubiquitin chain formation. These chains can form through linkage at one of ubiquitin’s seven lysine residues or its N-terminal methionine, or by attaching ubiquitin to additional lysines on the substrate protein. The specific site and linkage type dictate the fate of the modified protein (3).

## Spectrum of UBA1 mutations

**Figure 2:**
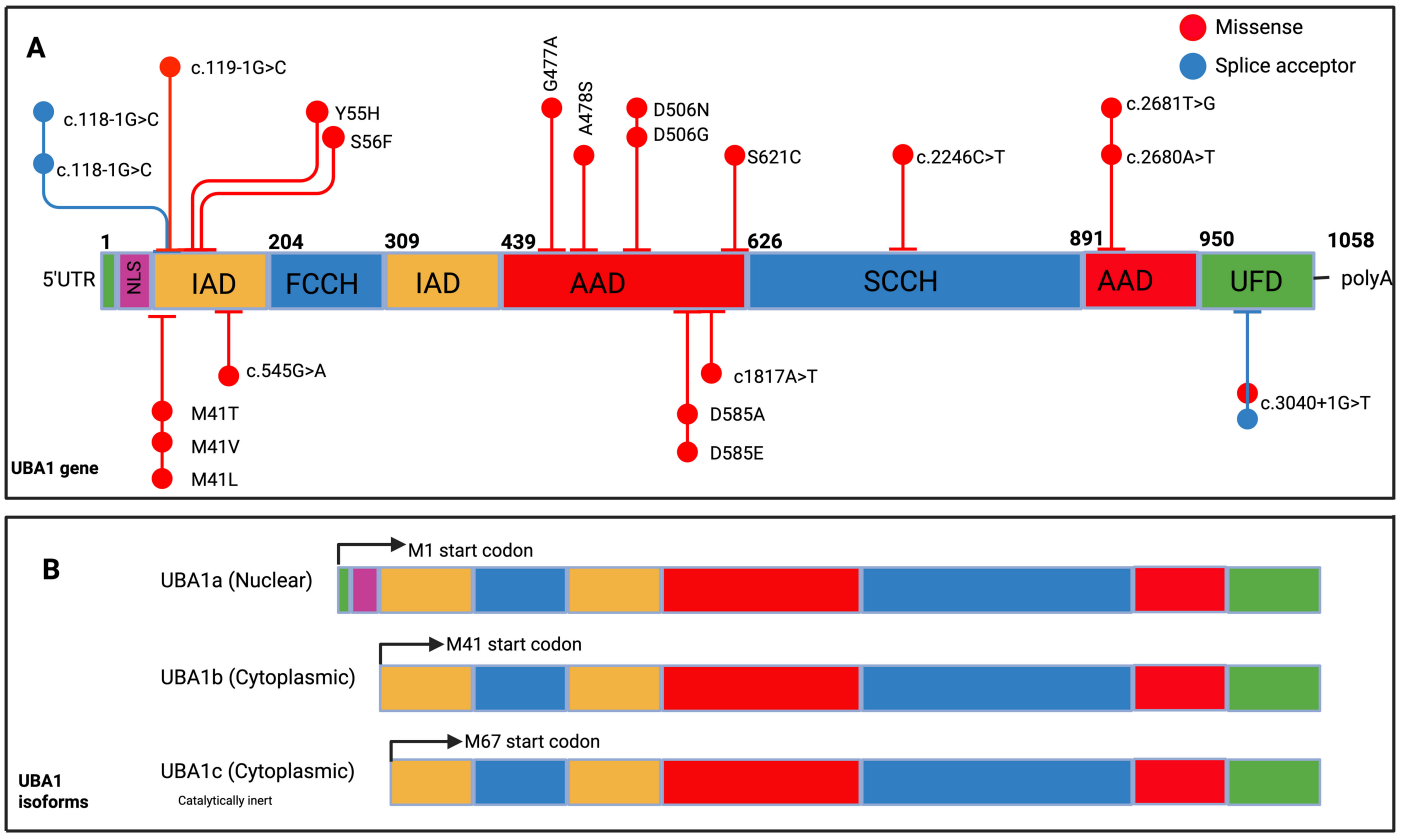
UBA1 gene structure, spectrum of mutations identified in VEXAS and UBA1 isoforms. **(A)** UBA1 gene structure, spanning amino acids 1 to 1058, with various functional domains highlighted in distinct colours. These include the nuclear localization signal (first 11 codons, NLS), the inactive adenylation domain (IAD), the first segment of the active catalytic cysteine (FCCH), the active adenylation domain (AAD), the second segment of the active catalytic cysteine (SCCH), and the C-terminal Ubiquitin Fold Domain (UFD). The diagram also marks representative mutations. **(B)** Two primary UBA1 isoforms: UBA1a, which begins at methionine 1 (M1, depicted in black), and UBA1b, starting at methionine 41 (M41, shown in green). Additionally, the non-canonical isoform UBA1c is displayed, initiating at methionine 67 (M67, indicated in dark blue). Modified with permission from Koster et al., *Am J Hematol*. (2024) 99(2):284-99.

VEXAS is driven by somatic mutations in the UBA1 gene, which normally produces two major isoforms: a nuclear isoform (UBA1a) and a cytoplasmic isoform (UBA1b), translated from distinct methionine start sites (4) (**Figure 2B**). The original VEXAS cohort identified recurrent missense mutations at p.Met41—including p.Met41Leu, p.Met41Val, and p.Met41Thr—each disrupting the AUG start codon at that position (4). This prevents production of UBA1b and instead leads to a truncated isoform, UBA1c, initiated at Met67, which lacks normal cytoplasmic function (5). *In vitro* studies suggest some residual UBA1b may still be produced through non-AUG initiation, though in reduced quantities (5).

The relative expression of UBA1 isoforms is tightly regulated during haematopoietic differentiation and cell cycling (6). In VEXAS, the loss of UBA1b and emergence of UBA1c alters this equilibrium, with residual UBA1b levels correlating with disease severity: p.Met41Val yields the lowest levels and worst outcomes, while p.Met41Leu supports higher residual activity and milder disease (5). Whether different mutations impact interactions with downstream E2/E3 enzymes remains unclear.

Larger cohort studies (5, 7) confirm genotype–phenotype correlations. Ferrada et al. (5) identified worse survival with p.Met41Val, while p.Met41Leu was associated with improved prognosis. Georgin-Lavialle et al. (7) similarly found no deaths with p.Met41Leu over 5 years, though longer follow-up in Ferrada et al. revealed poorer long-term outcomes with p.Met41Val (5).

Additional non-Met41 mutations have now been described, including splice variants and point mutations affecting regions shared by UBA1a/UBA1b, typically disrupting both isoforms without generating UBA1c (8, 9) These expanding genotypic profiles are summarized in **Figure 2**, with phenotypic correlations explored in depth in **Table 1**.

**Table 1:** Summary of spectrum of UBA1 mutations recorded in the literature with additional information on variant classification, recorded clinical phenotype and treatment outcome where available.

UBA1 mutation (cDNA → protein)	Variant class	Exon / domain	Core clinical phenotype / presentation	Reported outcome(s)	Reference
c.122T>C → p.Met41Thr	Missense	Exon 3 / initiation adenylation domain (IAD)	Most common VEXAS allele (~50 %); high-grade fevers, chondritis, Sweet-like dermatosis, macrocytic anaemia, vacuolated myeloid precursors	Five-year OS ≈ 83 % with supportive care; high steroid dependence	(4)
c.121A>G → p.Met41Val	Missense	Exon 3 / IAD	Second most common (~25 %); “undifferentiated” systemic inflammation; lowest residual UBA1b	Poorest Met41 survival (5-yr OS ≈ 63 %)	(5)
c.121A>C → p.Met41Leu	Missense	Exon 3 / IAD	~20 %; classic VEXAS with prominent Sweet’s lesions	Mildest Met41 course; French 5-yr OS ≈ 100 %	(7)
c.118-1G>C → p.?	Splice-site loss (acceptor)	Intron 2 / Ex 3 junction	Loss of exon 3, abolishing UBA1b; phenotype mirrors Met41 missense	Severity similar to Met41 Thr/Val; chronic, steroid-dependent	(10)
c.119G>C → p.Gly40Ala (in cis with p.Met41Leu)	Missense doublet	Exon 3 / IAD	Single case; partial rescue of UBA1b, slightly fewer flares	Alive; steroid-responsive	(5)
c.163T>C → p.Tyr55His	Missense	Exon 3 / IAD	Index 76-y male; ring-sideroblast MDS, Sweet’s dermatosis, anaemia	Corticosteroid-responsive; transfusion-free at 3 y	(11)
c.167C>T → p.Ser56Phe	Missense	Exon 3 / IAD	Thermolabile E1; milder systemic inflammation, scant vacuoles	Indolent on low-dose steroids; ≥ 5 y survival	(10)
c.545G>A → p.Arg182His	Missense	Exon 5 / IAD	Reported in ClinVar (t-MDS + VEXAS features)	Outcome unreported	ClinVar VCV002968676.2
c.1430G>C → p.Gly477Ala	Missense	Exon 14 / adenylation domain (AAD)	Severe neutrophilic vasculitis, anaemia without Met41 mutation	Allo-HSCT produced durable remission	(9)
c.1432G>T → p.Ala478Ser	Missense	Exon 14 / AAD	Single patient; cutaneous vasculitis, cytopenias, sparse vacuoles	Limited data; alive, steroid-dependent	(12)
c.1754_1755delinsGC → p.Asp585Ala	Missense	Exon 16 / AAD	Hypercellular MDS, mild vasculitis/arthralgia; co-clonal with p.Asp585Glu	Chronic, steroid-responsive; clones stable ≥ 18 mo	(13)
c.1755T>A → p.Asp585Glu	Missense	Exon 16 / AAD	Minor subclone in same patient as p.Asp585Ala	Same indolent course	(13)
c.1817A>T → p.Asn606Ile	Missense	Exon 17 / ubiquitin-binding interface III(Subclone)	9 % VAF subclone with dominant p.Ile894Ser; full VEXAS picture	Active disease; stepwise clonal evolution	(14)
c.1861C>G → p.Ser621Cys	Missense	Exon 18 / AAD	Six-patient series: skin nodules, arthritis, low-grade systemic signs; all MDS	Prognosis unclear (short follow-up)	(11)
c.2246C>T → p.Pro749Leu	Missense	Exon 20	Single 73-y male: transfusion-dependent anaemia, minimal inflammation	Insufficient follow-up (alive at report)	(15)
c.2680A>T → p.Ile894Phe	Missense	Exon 23 / ubiquitin-binding interface	Major 37 % VAF clone with minor p.Tyr55His; Sweet’s syndrome, anaemia	Corticosteroid-responsive; transfusion-independent	(10)
c.2681T>G → p.Ile894Ser	Missense	Exon 23 / ubiquitin-binding interface	Dominant 56 % VAF driver with p.Asn606Ile subclone; severe inflammation	Active disease; long-term outcome not yet published	(14)
p.Pro1014Leu + splice donor	Missense + splice loss	Exon 24 / intron 24 junction	Complex 3′ lesion; decades-long multi-organ autoimmunity before VEXAS-MDS	Highly refractory; multiple immunosuppressants; secondary malignancies	(15)

Female VEXAS cases are rare due to X-linked inheritance but can occur in settings of Turner syndrome, extreme X-inactivation skewing, or loss of the wild-type allele (16–18). Mutant clones are confined to hematopoietic cells, especially myeloid progenitors while lymphoid and non-hematopoietic cells remain wild-type (4). Germline UBA1 loss causes embryonic lethality or severe developmental syndromes (19).

Finally, somatic UBA1 mutations have been implicated in lung cancer in female never-smokers, suggesting wider oncologic relevance beyond VEXAS (20).

## UBA1-induced proteostasis failure, ER stress, and inflammasome activation

VEXAS syndrome has emerged as a paradigm for adult-onset autoinflammatory disease caused by somatic mutations that disrupt core cellular housekeeping. Acquired loss of function variants in the X-linked ubiquitin-activating enzyme UBA1 derail ubiquitination-dependent protein quality control, provoking endoplasmic reticulum stress and type I interferon–driven myeloid inflammation. The resulting clinical picture demonstrates how a single somatic lesion can ignite systemic innate-immune activation in otherwise immunocompetent adults (4, 21) (**Figure 1C**).

Unlike late-onset inflammatory disorders that arise from multifactorial immune dysregulation (e.g., giant-cell arteritis, polymyalgia rheumatica) or myelodysplastic-syndrome where clonal hematopoiesis predominates with inflammatory manifestations seen in only a subset (22). VEXAS couples autoinflammation and clonal hematopoiesis (4). Its characteristic cytoplasmic vacuoles, macrocytic anaemia, and steroid-refractory inflammation uniquely bridge rheumatology and haematology, underscoring the need to screen for somatic UBA1 mutations when confronted with late-onset, treatment-resistant inflammatory syndromes.

Pathogenic UBA1-loss variants interrupt the initiation step of ubiquitin conjugation, impairing endoplasmic reticulum (ER) associated degradation and allowing misfolded proteins to accumulate. Sustained unfolded-protein response signalling drives mitochondrial dysfunction, reactive oxygen-species production (ROS), Ca²^+^ influx and K^+^ efflux (23–25).

The unfolded protein response (UPR) consists of three primary signalling pathways, each initiated by a distinct sensor: inositol-requiring enzyme 1α (IRE1α), PRKR-like ER kinase (PERK), and activating transcription factor 6α (ATF6α). These sensors are all triggered by the accumulation of misfolded proteins. Activation of the UPR can directly stimulate various innate immune pathways, including NF-κB signalling, while activation of pathogen recognition receptors can, in turn, promote UPR activation (24–26).

Zebrafish- UBA1 models further illustrate this mechanism, and its further specificity to dysregulation of the proximal ubiquitylation cascade showing accumulation of the transcription factor IRF3 and excessive interferon production (27). Recent transcriptomic analyses of VEXAS patient biopsies demonstrate a prominent “type 1” immune signature characterized by upregulation of type I and II IFN-response genes, along with IL-1β and other cytokine signals (21, 28).

Similar proinflammatory cytokine signatures were recently observed by in an elegant xenograft mice model generated using a base-editing strategy generating UBA1 (p.Met41Thr) HSPCs (29).

ER stress in UBA1-mutant myeloid progenitors generate ROS, drive mitochondrial dysfunction and other danger signals that activate NF-κB, up-regulating pro-IL-1β and NLRP3 (28). Oxidised mitochondrial DNA further amplifies NLRP3 activation (30). These cues licence assembly of the canonical NLRP3 inflammasome (while a role for sensors such as NLRC4 remains unproven) (28). Downstream cleavage of gasdermin-D by caspase-1 or caspase-4/5/11 drives pyroptosis with sustained IL-1β/IL-18 secretion, linking the primary proteostasis defect to the febrile, neutrophil-predominant inflammation that characterises VEXAS (21).

Recent ubiquitin-biology studies underline how defective ubiquitin conjugation amplifies this cascade. Mishra et al. (31) demonstrated that the E2 enzyme UBE2L3 normally tags pro-IL-1β for K48-linked degradation; genetic or pharmacological loss of UBE2L3 stabilised the cytokine precursor, producing excess IL-1β and neutrophilic disease after NLRP3 activation Complementary work shows that mixed ubiquitination of NLRP3 by the gp78/Insig-1 E3 complex restrains sensor oligomerisation, so impaired ubiquitin charging likewise prolongs inflammasome stability (32).

Taken together, these data place the UBA1-ER-stress–inflammasome axis at the core of VEXAS pathogenesis and provide a rationale for therapeutic strategies that directly inhibit NLRP3, caspase-1, or downstream IL-1 signalling.

Although the inflammatory drive in VEXAS is primarily innate, secondary perturbations in the adaptive immune system also emerge. Chronic overproduction of interleukin-6 (IL-6) fosters T helper 17 (Th17) cell expansion and polyclonal hypergammaglobulinemia (21) potentially explaining the high rate of overlapping features with autoimmune conditions such as relapsing polychondritis or vasculitis (4, 5). Moreover, T cell repertoire analyses have demonstrated clonal expansions of cytotoxic CD8+ T cells in VEXAS, which exhibit cytotoxic gene signatures and produce interferon-γ (21). Whether these T cell expansions actively contribute to pathology by secreting interferon-γ that further activates macrophages or merely represent an epiphenomenon remains an open question.

## VEXAS as an acquired proteosomopathy

The proteasome is a multi-component enzyme complex essential for degrading proteins tagged with ubiquitin, allowing cells to eliminate misfolded or surplus proteins. It consists of a 20S catalytic core that provides proteolytic functions and is flanked by 19S regulatory particles. These regulatory units, made up of ‘base’ and ‘lid’ sub-complexes, are responsible for recognising and guiding ubiquitin-tagged proteins to the core for degradation. Although the proteasome is broadly expressed, its core structure can incorporate specialised subunits depending on tissue type, giving rise to variants such as immunoproteasomes (33).

Proteasome dysfunction in VEXAS shares mechanistic parallels with inherited biallelic loss-of-function mutations in proteasome-related genes. These mutations result in a subset of systemic autoinflammatory disorders known collectively as proteasome-associated autoinflammatory syndromes (PRAAS). These include conditions such as Nakajo–Nishimura syndrome and CANDLE syndrome (chronic atypical neutrophilic dermatosis with lipodystrophy and elevated temperature) (34) In these conditions, mutations in immunoproteasome subunits lead to a perpetual cycle of cytoplasmic waste protein accumulation and innate immune activation. Excess misfolded proteins provoke a constitutive type I interferon (IFN) response, which in turn further impairs proteasomal clearance, creating a feed-forward loop of chronic inflammation (34). CANDLE syndrome is now listed as a type I interferonopathy by the International Union of Immunological societies (35). Affected individuals typically exhibit early-onset fevers, skin rashes, neurological manifestations like intracranial calcifications, arthritis, and progressive lipodystrophy (34). Suggesting that both tissue-specific expression patterns and varying demands for proteasome activity in certain cell types may underlie the inflammatory phenotype, despite the proteasome’s fundamental role across most cellular functions.

Analogously, *UBA1* mutations in VEXAS likely engage type I IFN signalling pathways as part of the inflammatory cascade. Recent transcriptomic analyses of VEXAS patient biopsies demonstrate a prominent “type 1” immune signature characterized by upregulation of type I and II IFN-response genes (21).

Such findings underscore the convergence of UPR driven stress and innate immune sensors (e.g. cGAS–STING or RIG-I-like receptors) (36) that can link proteostatic stress to interferon pathways. Therapeutically, this is supported by reports of VEXAS patients responding to JAK inhibitors (37).

Importantly, VEXAS arises in late adulthood, potentially suggesting that age-related declines in proteostasis contribute to its pathogenesis. Proteostasis capacity wanes with advancing age, leading to accumulation of damaged proteins, basal NF-κB activation, and a pro-inflammatory milieu (33). A so call state of “Inflammaging”.

As an individual ages they experience progressive erosion of cellular proteostasis, with waning of chaperone capacity, autophagic flux slows and the unfolded-protein response (UPR) loses efficiency, permitting chronic endoplasmic-reticulum stress and the build-up of misfolded proteins (33, 38, 39).

When a somatic UBA1 loss-of-function mutation arises on this background, ubiquitin charging becomes rate-limiting, further crippling ER-associated degradation and accelerating DNA-damage and oxidative-stress signalling in haematopoietic and myeloid progenitors. Single-cell and proteomic studies of VEXAS marrow show concordant UPR activation and STAT1-driven inflammatory gene expression, indicating that many mutant cells enter a senescent state and elaborate a senescence-associated secretory phenotype (SASP) rich in IL-6, IL-1β and IL-18 (40). This convergence of age-related proteostasis decline and UBA1 insufficiency therefore provides a mechanistic basis for the persistent, cytokine-amplified inflammation that typifies VEXAS syndrome in older adults.

Epigenetic drift compounds these effects. Ageing haematopoietic stem cells (HSCs) acquire DNA-methylation and chromatin-accessibility changes that silence lymphoid-associated enhancers while preserving or activating myeloid programmes, producing the well-described myeloid skewing of elderly marrow (41).

UBA1-mutant clones emerge within this permissive epigenetic landscape; the inflammatory SASP milieu reinforces myeloid-biased transcription through NF-κB and STAT signalling and further remodels chromatin at myeloid loci. The result is clonal dominance of UBA1-mutant myeloid cells with impaired erythroid, megakaryocytic and lymphoid output, a pattern already evident at the stem-cell level in patient samples (28).

Morphologically, ultrastructural studies reveal that the bone marrow vacuoles characteristic of VEXAS contain lipid droplets and disorganised organelles—such as mitochondrial fragments—indicating incomplete clearance of cellular constituents (42). This, in turn, suggests that the autophagy-lysosome pathway is upregulated to compensate for defective proteasomal degradation, sequestering undegraded material into vacuoles while persistent protein aggregation continues to fuel cellular stress.

Autophagy defects have been identified as a key contributor to a variety of neurodegenerative diseases. With cellular aging impact on lysosomes and autophagy hypothesised to serve as a tipping point for the late-age emergence of neurodegenerative disorders (43, 44).

Thus, VEXAS can be viewed as an acquired, age-intensified proteasomeopathy in which somatic UBA1 dysfunction meets declining proteostatic capacity and epigenetic myeloid bias, igniting a self-perpetuating circuit of proteotoxic stress, type I-interferon-driven autoinflammation, and clonal myeloid dominance.

## Clonal dominance

In many VEXAS patients, age-related “background” clonal haematopoiesis (CH) coexists, studies show ~60% have additional mutations in genes with primary functions as epigenetic regulators, such as DNMT3A or TET2 (45, 46). Interestingly, the *UBA1*-mutant clone usually remain dominant even when typical CH mutations are present. Several cases illustrate competition or coexistence of *UBA1*-mutant clones with other hematologic clones. One patient with long-standing Calreticulin (CALR)-mutated essential thrombocythemia (ET) later acquired a *UBA1* mutation; the *UBA1*-mutant clone progressively overtook the CALR-mutant clone, extinguishing the ET phenotype as VEXAS features emerged (47). This clonal sweep underscores the strong selective advantage of *UBA1* mutations, capable of overcoming even a vigorous myeloproliferative driver. Another report described a male with concurrent chronic myeloid leukaemia (CML, BCR-ABL1 clone) and VEXAS. At CML diagnosis, the BCR-ABL1 clone dominated (with the *UBA1* allele burden falling), but after tyrosine kinase inhibitor therapy suppressed CML, the *UBA1*-mutant clone re-expanded and the inflammatory symptoms of VEXAS recrudesced (48). These unique cases demonstrate that clonal dominance in VEXAS may depend on the relative competitive advantage of coexisting neoplastic clones and driver mutations, with the *UBA1* clone flourishing when a more proliferative neoplastic clone or signalling pathway mutation is absent or controlled. Intriguingly, independent *UBA1* mutant clones can arise in a single individual: a recent case detailed three distinct somatic *UBA1* mutations (each defining a separate clone) in one VEXAS patient (14).

The microenvironment of VEXAS is characterized by chronic inflammation (elevated cytokines such as IL-6, IL-1β, interferon signalling) and often by consequences of prior immunosuppressive therapy, creating unique cell-extrinsic selective pressures. Inflammatory stress favours *UBA1*-mutant clone dominance in several ways. Clonal dominance has often been ascribed to cell-intrinsic expansion capacity caused by proliferative advantage and enhanced self-renewal. However, recent data challenge this hypothesis by demonstrating that the establishment of a cell-extrinsic inflammatory milieu by UBA1-mutant clones relatively more harmful to wild-type than to mutant cells in VEXAS syndrome (29). Wild-type HSPCs exposed to the inflammatory environment in VEXAS were noted to upregulate of inflammatory responses and activate proapoptotic and p53 target genes, in addition to impaired clonogenicity (29).

The resilience of UBA1-mutant cells to this ‘poisonous’ extracellular environment may be acquired through states of dormancy or undergoing senescence, which may protect UBA1-mutant cells from inflammation-induced apoptosis and immune clearance (29, 45, 46). IFN signature was noted to be lower in UBA1-mutant cells compared to wild-type HSPCs indicating that UBA1-mutant progenitors might be less responsive to inflammatory stimuli (49). Additionally, a recent single cell study (50) indicated that activation of the UPR in UBA1 mutated cells resulted in activation of an anti-apoptosis pathway PERK providing a key survival mechanism to UBA1 p.Met41Val/Thr mutated HSPCs.

Moreover co-mutations like DNMT3A (frequently co-occurring in VEXAS) may also act to confer resilience to inflammatory cytokine-induced apoptosis in hematopoietic stem cells, giving the combined *UBA1*–DNMT3A clone a fitness edge under pro-inflammatory conditions (51). Experimental and clinical data suggest that hematopoietic cells with DNMT3A mutations resist interferon-γ and TNF-α–mediated differentiation or death, thereby surviving (or even expanding) amidst inflammation​ (51) This likely synergizes with the *UBA1* mutation’s effects.

The relative immunodeficiency in VEXAS (due to cytopenia’s and dysfunctional lymphocytes) means less immune surveillance to curtail clonal proliferation. Indeed, treatments often involve corticosteroids or other immunosuppressants, which while alleviating inflammation, might reduce immune-mediated control of the clone, potentially allowing the UBA1 mutant population to persist.

Clonal selection in VEXAS reflects a “survival of the fittest” paradigm in the bone marrow. As noted, UBA1-mutated clones gain dominance through intrinsic advantages (hyporesponsiveness to inflammatory and pro-apoptotic signals, and stress tolerance) and extrinsic conditions (chronic inflammation, an altered immune environment) that favour their survival. These clones can co-opt the inflammatory environment to their benefit, outgrow coexistent hematologic clones, and dictate the clinical course. Given the mechanisms of clonal dominance in VEXAS, a multi-pronged treatment strategy combining anti-inflammatory and clone-eradicating treatment aiming at resetting the inflammatory milieu and simultaneously depleting the mutant clones would be required to prevent progression to bone marrow failure and irreversible end organ insults.

Despite the progress made to understand disease pathogenesis in VEXAS, the following observations remain unclear:

1. mechanisms of myeloid skewing, senescence and hypo-responsiveness to inflammatory milieu of UBA1-mutant HSPCs cells.
2. Enhanced egression of HSPCs from the bone marrow.
3. Very low rates of clonal progression to secondary acute myeloid leukaemia in patients with VEXAS syndrome despite the high prevalence of MDS and co-occurrence of CHIP mutations.

## Clinical spectrum of VEXAS

VEXAS syndrome presents as a late-onset, multisystem disorder that bridges autoinflammatory and hematologic disease, with a median age of onset around 66 years (52). Hematologically, patients typically exhibit macrocytic anemia and thrombocytopenia, which may become transfusion-dependent in severe cases. Recent work by Rodrigues et al. (53) has demonstrated that UBA1 mutations impair erythropoiesis by inducing early apoptosis in erythroid progenitors via p53 activation and ribosomal stress, a mechanism reminiscent of Diamond–Blackfan anemia. In contrast, myeloid differentiation remains largely intact, allowing for persistent neutrophil-driven inflammation despite only mildly reduced or normal neutrophil counts. Importantly, mature erythroid cells in VEXAS derive predominantly from residual UBA1-wildtype progenitors, and anemia severity reflects the functionality of this wildtype compartment (53).

Systemically, most patients experience constitutional symptoms such as fevers, weight loss, and fatigue during inflammatory flares (54, 55). Dermatologic involvement is frequent, presenting as neutrophilic dermatoses or leukocytoclastic vasculitis (56) often leading to initial misdiagnosis. Articular and cartilaginous involvement is also common: many fulfil criteria for relapsing polychondritis—a condition now recognized to include a subset of VEXAS cases—and around 50% present with arthritis ranging from episodic polyarthritis to erosive arthropathy (4, 54, 55). Some individuals display features overlapping with systemic vasculitides, such as polyarteritis nodosa or granulomatosis with polyangiitis (57). Pulmonary manifestations are seen in over half of patients and include interstitial lung disease, organizing pneumonia, or inflammatory infiltrates, though infections must also be considered (58). Inflammatory eye disease—such as scleritis, episcleritis, or uveitis—may occur, reflecting the syndrome’s multisystem inflammatory profile (59).

Although phenotypic expression varies, most cases combine hematologic abnormalities and rheumatologic or inflammatory features. Definitive diagnosis requires molecular confirmation of a somatic UBA1 mutation through targeted or exome sequencing of blood or marrow cells.

## Therapeutic approaches in VEXAS syndrome

The aforementioned clinical features of VEXAS syndrome rarely occur in the same combination or temporal sequence. Large registry and case-series analyses therefore describe highly variable baseline disease burdens and organ-system weights, even before therapy is considered (4, 5, 7, 54, 55). This biological heterogeneity translates directly into disparate treatment outcomes, for example; high-dose glucocorticoids achieve rapid symptom control in most patients yet relapse rates differ markedly once doses fall below 10 mg/day, while responses to azacitidine, JAK-STAT inhibitors or IL-1 blockade are strongly influenced by the presence of concurrent MDS, specific UBA1 variants and the predominance of cutaneous versus cartilaginous disease (60).

Inter-study comparisons are further complicated by inconsistent outcome nomenclature. In the French national targeted-therapy cohort (7), “complete response” (CR) required clinical quiescence, C-reactive protein (CRP) ≤ 10 mg L^-^¹ and ≤ 10 mg/day prednisone, whereas “partial response” (PR) meant clinical quiescence plus ≥ 50% reductions in both CRP and steroid dose (7). The azacitidine registry (61) adopted a similar biochemical threshold but added the absence of any other immunosuppressant for CR, while other retrospective series define PR solely by physician judgement or steroid-sparing ≥ 20%. A recent Lancet Rheumatology commentary highlighted that such divergent criteria yield reported CR rates from 0% to 66% for the same intervention, undermining meta-analysis and evidence-based guidelines (62).

Future studies should therefore implement a harmonised, domain-based outcome frameworks in order to enable would enable robust cross-cohort comparisons, facilitate pooled analyses and accelerate the rational development of targeted therapies for this heterogeneous syndrome.

## Glucocorticoids and the need for steroid-sparing therapies

Initial management of VEXAS syndrome often relies on high-dose glucocorticoids due to their prompt anti-inflammatory effect. Patients frequently show clinical improvement with corticosteroids, but this approach is non-curative and accompanied by significant toxicity (including diabetes, infections, osteoporosis, and cardiovascular events) upon long-term use. Consequently, a central goal is to taper steroids by introducing steroid-sparing therapies that control disease activity (63). Over time, most patients require multiple sequential treatments; in one series of 59 genetically confirmed VEXAS patients treated with 71 targeted therapies, many received three to five different agents before considering definitive options like transplant (7).

## Interleukin-1 blockade

Targeting IL-1 has emerged as a therapeutic strategy given the autoinflammatory nature of VEXAS. Anakinra (an IL-1 receptor antagonist) is the most commonly used agent in this category. Clinical reports and case series indicate that IL-1 inhibition can alleviate symptoms and reduce inflammatory markers in a substantial subset of patients (52, 64). A recent systematic review found partial clinical responses (≥ 40% reduction in disease severity) in roughly 47% of patients treated with anti-IL-1 therapy, though complete remissions were achieved in only ~ 13% (64). These data suggest anakinra often provides meaningful improvement and steroid-sparing effects, but rarely induces full remission as monotherapy. Anakinra’s safety profile is generally acceptable; however, injection-site reactions are notably frequent (reported in over half of patients in some cohorts) (64). Overall, IL-1 blockade is considered a valuable option in VEXAS, especially for managing fevers, pain, and other inflammatory symptoms, and it is often used early given its rapid onset of action and clinician familiarity.

## Interleukin-6 inhibition

IL-6 is another key cytokine driving inflammation in VEXAS. IL-6 receptor monoclonal antibodies (tocilizumab and sarilumab) have been used off-label in VEXAS patients with encouraging partial responses. Pooled data from recent studies indicate that IL-6 inhibitors yield partial responses in roughly 70% of patients (65). Approximately one-quarter of patients achieve complete clinical remission on IL-6 blockade (65). These agents can reduce fever, skin lesions, and acute phase reactants, helping to taper steroids in a number of cases. Despite these benefits, relapses are common when IL-6 therapy is used alone, and not all patients respond. IL-6 inhibitors are thus considered a second-line or adjunct therapy in many centres. Notably, a comparative analysis suggested that while IL-6 and IL-1 targeted treatments are both active in VEXAS, their efficacy may be somewhat lower than that observed with Janus kinase inhibitors (65). Nonetheless, IL-6 blockade remains an important option for patients who cannot tolerate other therapies or in whom IL-6–mediated symptoms (e.g., systemic inflammation, anemia) are prominent (65).

## Janus kinase inhibitors (ruxolitinib and others)

JAK inhibitors, especially ruxolitinib, have become a leading therapy for VEXAS syndrome. In a multicentre retrospective study of 116 patients, ruxolitinib achieved a complete remission rate of 42% and partial improvement in 79% of cases, outperforming tofacitinib and baricitinib (37, 66, 67). The rapid onset of action permits steroid tapering, and some patients sustain disease control on ruxolitinib monotherapy over extended follow-up (67). Adverse effects include cytopenias, infections, and an elevated thrombotic risk, mirroring known JAK inhibitor profiles (37). Careful monitoring is required, but overall the benefit–risk balance favors ruxolitinib for many patients.

## Hypomethylating agents

For those with concomitant myelodysplastic features or clonal progression, hypomethylating agents such as azacitidine may reduce UBA1-mutant burden and improve cytopenias. In a cohort of 36 VEXAS patients treated with azacitidine, 25% achieved a complete response and 39% a partial response at 12 months, with mean corticosteroid dose reduced by 40% (65). A small case series reported sustained clinical and molecular remission after discontinuation of azacitidine in two patients, suggesting potential eradication of the mutant clone (68).

## Other medical therapies

Anti–TNF-α inhibitors (e.g., infliximab, adalimumab) have been used in individual cases but with low complete response rates (< 10%) (69). Conventional immunomodulators; methotrexate, cyclophosphamide, calcineurin inhibitors have shown inconsistent and typically transient benefits in refractory VEXAS (7).

## Allogeneic hematopoietic stem cell transplantation

Allo-HSCT remains the only potentially curative therapy for VEXAS, aiming to eliminate the UBA1-mutant clone and reconstitute normal hematopoiesis. In a review of seven transplanted patients, 86% achieved complete remission of inflammatory and hematologic manifestations, with one transplant-related death (14%) at a median follow-up of five months (70). Treatment-related mortality in VEXAS HSCT series appears lower than historical myeloid transplant cohorts (~ 25%), but long-term relapse rates are not yet defined (7, 70). Patient selection and reduced-intensity conditioning regimens are critical to optimize outcomes.

**Figure 3:**
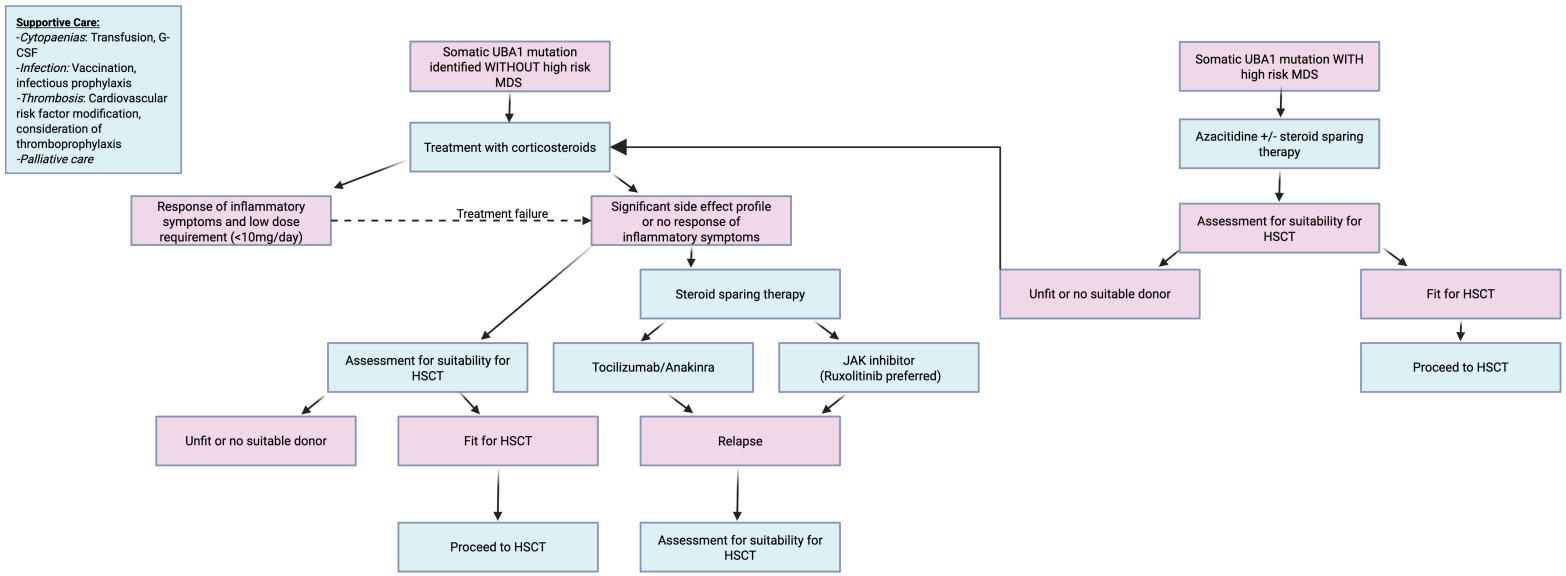
Proposed treatment algorithm for VEXAS best on best available current data.

**Figure 3** delineates a proposed algorithmic management strategy for VEXAS patients based on best available current data.

## Experimental and targeted approaches

Preclinical investigations are exploring proteasome modulators, unfolded protein response inhibitors, and gene-editing strategies targeting the UBA1 mutation.

**Figure 4:**
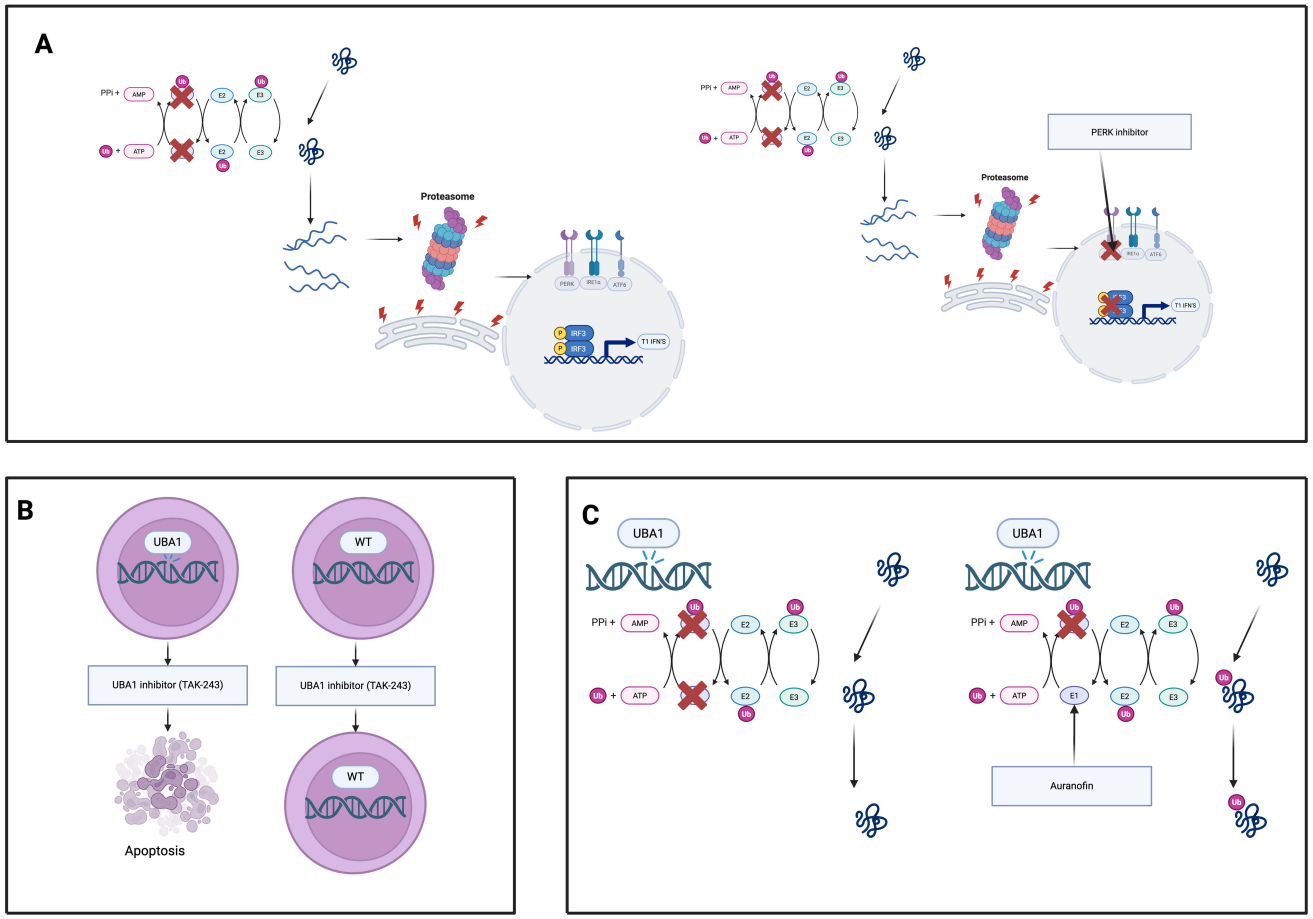
Experimental therapeutic strategies targeting UBA1-mutant clones in VEXAS syndrome. **(A)** UBA1 inhibitors (e.g., TAK-243) selectively induce apoptosis in UBA1-mutant hematopoietic cells while sparing wild-type cells, exploiting their dependency on residual UBA1 activity. **(B)** Proteostasis modulators (e.g., PERK inhibitors) disable the unfolded protein response, sensitizing UBA1- mutant cells to proteotoxic stress and promoting cell death. **(C)** Auranofin enhances residual UBA1 enzymatic activity, restoring ubiquitination, reducing proteotoxic stress, and dampening inflammation in UBA1-mutant cells.

**Figure 4** summarises 3 experimental classes of therapeutics.

## UBA1 inhibitors

TAK-243 (also known as MLN7243) is a first-in-class small-molecule inhibitor of ubiquitin-activating enzyme UBA1 that has shown selective toxicity toward UBA1-mutant cells. In a VEXAS syndrome model using a myeloid cell line engineered with a UBA1 Met41euL mutation, TAK-243 treatment led to preferential apoptosis and loss of the UBA1-mutant cells compared to wild-type, indicating that reduced UBA1 activity renders cells uniquely vulnerable to UBA1 inhibition (71). Consistently, primary CD34+ hematopoietic progenitor cells from patients with VEXAS were found to be highly sensitive to TAK-243. VEXAS patient CD34+ cells exhibited significantly lower IC_50 values and higher rates of apoptosis upon TAK-243 exposure than cells from healthy donors or myelodysplastic syndrome (MDS) controls, suggesting a broad therapeutic window in which UBA1-mutant clones are selectively targeted while sparing normal hematopoiesis (72). These findings provide a strong rationale for UBA1 inhibitors like TAK-243 as targeted therapy against the UBA1-mutant hematopoietic clone in VEXAS syndrome.

## Proteostasis modulators

Cells carrying UBA1 mutations display chronic activation of the unfolded protein response (UPR), particularly through the protein kinase R-like ER kinase (PERK)–ATF4 signaling arm of the UPR (50). This adaptive stress response appears to confer a survival advantage to UBA1-mutant myeloid cells by helping them cope with proteotoxic stress (50). Accordingly, pharmacological inhibition of the PERK pathway has been shown to preferentially induce death in UBA1-mutant cells. In experimental models, the selective PERK inhibitor GSK2606414 triggers significantly more apoptosis in UBA1-mutant hematopoietic cells than in wild-type cells (6, 50). In contrast, UBA1-mutant cells are not as reliant on wild-type UBA1 function and thus are uniquely susceptible when this pro-survival UPR signalling is blocked. Inhibiting the other UPR branches may have similar therapeutic effects: targeting IRE1α or ATF6 pathways is expected to disrupt the UPR-driven survival signals in UBA1-mutant cells, potentially reducing their fitness. Therefore, PERK inhibitors, and possibly IRE1α or ATF6 inhibitors, represent promising strategies to eliminate VEXAS clones by disabling the UPR-mediated proteostasis benefits on which UBA1-mutant cells depend.

## Auranofin (UBA1 reactivation)

Auranofin, an orally administered gold compound long used in rheumatoid arthritis, has emerged as a novel UBA1-reactivating agent. Recent studies identified auranofin as a potent enhancer of UBA1 enzymatic activity (73, 74). Mechanistically, auranofin binds to the ubiquitin-fold domain of UBA1 (forming a covalent adduct at cysteine-1039) and facilitates ubiquitin transfer from UBA1 to downstream E2 conjugating enzymes (73). In cells, auranofin’s enhancement of UBA1–E2 thioester formation leads to increased global protein ubiquitination and restoration of multiple ubiquitin-dependent processes that are impaired by UBA1 mutations. Notably, auranofin has been shown to promote the ubiquitination and proteasomal degradation of misfolded proteins (e.g. via improving ER-associated degradation), thereby relieving proteotoxic stress at nanomolar concentrations (73). By partially restoring UBA1 function in VEXAS-mutant cells, auranofin may reduce the downstream consequences of ubiquitination dysfunction. Alleviating the accumulation of ubiquitin-tagged substrates should dampen UPR overactivation and the resultant inflammatory cascade. Consistent with its known anti-inflammatory effects, auranofin and related gold compounds have been shown to suppress pro-inflammatory cytokine production (such as IL-1β and TNF-α) in activated macrophages (74). Thus, by reactivating residual UBA1 activity, auranofin may improve proteostasis and mitigate the aberrant inflammatory cytokine milieu in VEXAS syndrome. Importantly, the doses of auranofin required to enhance UBA1 are reported to be well below those historically used for arthritis, suggesting a feasible therapeutic index (6). This approach of pharmacologically boosting UBA1 function represents a promising avenue to restore normal ubiquitin signalling and quell inflammation in UBA1-mutant disease.

At present, all three approaches remain squarely investigational. TAK-243 is confined to early dose-escalation studies in relapsed/refractory AML, MDS and solid tumours with no VEXAS-specific trial yet open, making routine clinical use improbable in the next few years (75). Next-generation PERK/ISR inhibitors such as HC-5404 have only just completed first-in-human phase 1a safety studies in advanced solid tumours and have not been tested in clonal inflammatory disorders, so their application to VEXAS will likewise remain experimental until larger efficacy cohorts are undertaken (76, 77). Auranofin, although already licensed for rheumatoid arthritis and explored in phase II oncology trials, has so far been assessed for UBA1 re-activation only in pre-clinical systems, with no registered VEXAS trial; its off-label use may occur on a compassionate basis, but broad adoption awaits formal efficacy and dosing studies (6).

## Current knowledge gaps

First, the cell-intrinsic mechanisms that let UBA1-mutant haematopoietic stem and progenitor cells (HSPCs) out-compete wild-type neighbours remain unresolved. Recent single-cell and xenograft work shows that mutant HSPCs adopt a senescence-like, inflammation-resistant programme and rely on chronic PERK-ATF4 signalling to survive proteotoxic stress, yet it is still unclear how this state drives the characteristic myeloid skewing and relative lymphoid failure, or why only some E2/E3 pathways are selectively crippled. Mapping the full spectrum of ubiquitin-dependent processes lost (or rewired) downstream of partial UBA1 deficiency—and defining which arms of the unfolded-protein response are truly “druggable” without harming normal haematopoiesis—remains a high priority (6, 29).

Second, the rules that govern clonal architecture and malignant evolution are poorly defined. UBA1 mutations frequently coexist with canonical CHIP drivers such as DNMT3A or TET2, yet progression to overt AML is remarkably rare. Why inflammatory pressure confers a competitive advantage to UBA1-mutant clones but not to typical CHIP clones, and how additional lesions (or their order of acquisition) influence cytopenias, organ tropism, and survival, are open questions. Longitudinal studies integrating single-cell genomics with micro-environmental profiling are needed to explain why some patients remain stable while others develop marrow failure or plasma-cell dyscrasias (29, 45).

Third, translational progress is hampered by heterogeneous outcome definitions and a lack of prospective, biomarker-guided trials. Most therapeutic evidence still comes from small, retrospective series that use incompatible response criteria, making cross-study comparison and therefore rational trial design challenging. No validated disease-activity or flare score exists, patient-reported outcomes are rarely captured, and predictors of response to clone-directed agents (e.g., azacitidine, PERK inhibitors, TAK-243) or to immunomodulators remain speculative. Harmonising clinical endpoints, embedding molecular minimal residual disease assays, and prospectively collecting quality-of-life data are essential steps before experimental strategies such as UPR blockade, UBA1 re-activation, or early allogeneic transplant can be tested rigorously (78).

## Conclusion

VEXAS syndrome has rapidly evolved from an enigmatic autoinflammatory disorder into a prototype disease that links defective ubiquitin biology, proteostasis collapse and clonal haematopoiesis. Over the past five years, studies spanning structural biochemistry, single-cell genomics and *in vivo* modelling have clarified how somatic UBA1 loss derails ER-associated degradation, fuels a type I/II interferon milieu and drives senescence-like, myeloid-skewed haematopoiesis (6, 23, 29, 40). These insights place VEXAS on a mechanistic continuum with both autoinflammatory syndromes and age-related clonal haematopoiesis, underscoring its value as a clinical “Rosetta stone” for studying inflammation at the interface of innate immunity and stem-cell fitness. At the bedside, recognition of pathognomonic macrocytic anaemia, cytoplasmic vacuoles and steroid-refractory inflammation has improved diagnostic speed, while retrospective cohorts have provided a first therapeutic hierarchy; glucocorticoids for initial control, JAK–STAT blockade and IL-6 inhibition for steroid-sparing, azacitidine when marrow dysplasia predominates, and allogeneic HSCT as the only proven curative option (52, 60, 65, 78).

Yet the field now stands at an inflection point. Targeted agents that either disable stress-adaptation pathways (PERK/ISR inhibitors) or restore ubiquitin charging (UBA1 activators) have shown clone-selective toxicity in pre-clinical systems. To translate these advances, future trials must embed harmonised, domain-based response criteria, integrate molecular minimal-residual-disease monitoring and capture patient-reported outcomes so that efficacy signals can be compared across centres and therapies (62). Equally pressing is the need to unravel why UBA1-mutant HSPCs resist inflammatory apoptosis, how co-mutations such as DNMT3A modulate disease course, and why malignant transformation remains rare despite profound marrow stress. By aligning mechanistic discovery with rigorously designed clinical studies, the next decade should convert VEXAS from a disease framed by therapeutic gaps into one guided by precision, clone-directed interventions.
