# Correction: VEXAS: A review of current understandings and emerging treatment strategies

**DOI:** 10.3389/fimmu.2026.1807170

**Published:** 2026-02-27

**Authors:** Robert Holden, Yogeshraj Jeelall, Andrew McLean-Tooke, Kylan Pathmanathan, David Nolan

**Affiliations:** 1 Clinical Immunology, Royal Perth Hospital, Perth, WA, Australia; 2 Haematology, Royal Perth Hospital, Perth, WA, Australia; 3 Medical School, University of Western Australia, Perth, WA, Australia; 4 Clinical Immunology, Sir Charles Gairdner Hospital, Perth, WA, Australia; 5 Rheumatology, Sir Charles Gairdner Hospital, Perth, WA, Australia

**Keywords:** VEXAS syndrome, VEXAS, autoinflamatory diseases, myeloid cells, myelodyslastic syndromes

Following publication of this article, concerns were raised regarding the reuse of copyright-restricted material in **Figure 2**.

**Figure 2** was modified from **Figure 1** of Koster et al., originally published under an all-rights-reserved copyright. The authors have now obtained permission from the publisher (Wiley) for reuse of the modified figure. The corrected caption of **Figure 2** appears below:

“UBA1 gene structure, spectrum of mutations identified in VEXAS and UBA1 isoforms. (A) UBA1 gene structure, spanning amino acids 1 to 1058, with various functional domains highlighted in distinct colours. These include the nuclear localization signal (first 11 codons, NLS), the inactive adenylation domain (IAD), the first segment of the active catalytic cysteine (FCCH), the active adenylation domain (AAD), the second segment of the active catalytic cysteine (SCCH), and the C-terminal Ubiquitin Fold Domain (UFD). The diagram also marks representative mutations. (B) Two primary UBA1 isoforms: UBA1a, which begins at methionine 1 (M1, depicted in black), and UBA1b, starting at methionine 41 (M41, shown in green). Additionally, the non-canonical isoform UBA1c is displayed, initiating at methionine 67 (M67, indicated in dark blue). Modified with permission from Koster et al., *Am J Hematol*. (2024) 99(2):284–99.”

These corrections relate solely to figure licensing and attribution and do not affect the scientific content, interpretation, or conclusions of the article.

**Figure 3** was modified from Extended Data **Figure 7** of Molteni et al., which was published under a CC BY-NC-ND 4.0 licence that does not permit modifications. To ensure compliance with the licence terms **Figure 3** has been removed from the manuscript.

These corrections relate solely to figure licensing and attribution and do not affect the scientific content, interpretation, or conclusions of the article.

